# Drivers of litter mass loss and faunal composition of detritus patches change over time

**DOI:** 10.1002/ece3.7787

**Published:** 2021-06-23

**Authors:** Franziska K. Seer, Gregor Putze, Steven C. Pennings, Martin Zimmer

**Affiliations:** ^1^ Institute for Ecosystem Research Kiel University Kiel Germany; ^2^ Zoologisches Institut Christian‐Albrechts‐Universität zu Kiel Kiel Germany; ^3^ Department of Biology and Biochemistry University of Houston Houston TX USA; ^4^ Leibniz‐Centre for Tropical Marine Research Bremen Germany; ^5^ Faculty 02 Biology/Chemistry University Bremen Bremen Germany

**Keywords:** decomposition, habitat comparison, leaf litter, litter fauna

## Abstract

Decomposition of vegetal detritus is one of the most fundamental ecosystem processes. In complex landscapes, the fate of litter of terrestrial plants may depend on whether it ends up decomposing in terrestrial or aquatic conditions. However, (1) to what extent decomposition rates are controlled by environmental conditions or by detritus type, and (2) how important the composition of the detritivorous fauna is in mediating decomposition in different habitats, remain as unanswered questions. We incubated two contrasting detritus types in three distinct habitat types in Coastal Georgia, USA, to test the hypotheses that (1) the litter fauna composition depends on the habitat and the litter type available, and (2) litter mass loss (as a proxy for decomposition) depends on environmental conditions (habitat) and the litter type. We found that the abundance of most taxa of the litter fauna depends primarily on habitat. Litter type became a stronger driver for some taxa over time, but the overall faunal composition was only weakly affected by litter type. Decomposition also depends strongly on habitat, with up to ca. 80% of the initial detrital mass lost over 25 months in the marsh and forest habitats, but less than 50% lost in the creek bank habitat. Mass loss rates of oak versus pine litter differed initially but converged within habitat types within 12 months. We conclude that, although the habitat type is the principle driver of the community composition of the litter fauna, litter type is a significant driver of litter mass loss in the early stages of the decomposition process. With time, however, litter types become more and more similar, and habitat becomes the dominating factor in determining decomposition of older litter. Thus, the major driver of litter mass loss changes over time from being the litter type in the early stages to the habitat (environmental conditions) in later stages.

## Graphical abstract

Decomposition of vegetal detritus is one of the most fundamental ecosystem processes. In complex landscapes, the fate of litter of terrestrial plants may depend on whether it ends up in decomposition of terrestrial or aquatic conditions. While litter type is a significant driver of litter mass loss in the early stages of decomposition, litter types become more and more similar over time, and habitat becomes the dominating factor in determining the decomposition of older litter.

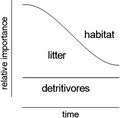

## 1 INTRODUCTION

The leaf litter of terrestrial trees can end up in multiple habitats with different abiotic conditions and faunas. In coastal ecosystems, for instance, litter may fall into terrestrial habitats, into high marsh habitats that are periodically inundated with seawater, or into creeks that are periodically inundated with freshwater. Thus, litter from a single tree might experience very different fates with regard to decay and decomposition. This matters because decomposition is one of the most fundamental ecosystem processes, transforming dead organic matter into freely available inorganic nutrients and providing the basis for essentially all nutrient cycles.

Decomposition is mediated by a complex network of interactions between microbes and detritivorous animals that, in turn, are controlled by environmental conditions, litter abundance and traits, and predators (for summary: Zimmer, 2019). For example, Treplin and Zimmer (2012) demonstrated that decomposition processes in aquatic versus terrestrial systems diverge fundamentally. In terrestrial systems, temperature and moisture strongly affect decomposition rates (Cisneros‐Dozal et al., 2007; Ise & Moorcroft, 2006). Species‐specific decomposition rates of different detrital (leaf litter) sources have been repeatedly documented, with variation in decomposition depending on chemical and physical characteristics of the leaves (for review: Gessner et al., 2010). However, leaf litter chemistry changes over time owing to leaching of soluble, and microbial decay of readily available, litter compounds (Nykvist, 1962; Schofield et al., 1998; Zimmer, 2002), and when submerged in water, species‐specific characteristics of different litter types converge within weeks (Treplin & Zimmer, 2012). Because of this convergence, it is likely that the relevance of the litter type, that is, different physicochemical characteristics, as predictors of the decomposition processes will decrease over time.

The role of the detritivorous fauna in decomposition has been debated. By shredding, consuming, and translocating detritus, macro‐detritivores accelerate microbial decay of detrital matter and nutrient cycling, and also directly contribute to detrital mass loss (Seeber et al., 2006; Treplin & Zimmer, 2012). Meso‐detritivores, by contrast, predominantly act as grazers on detrital surfaces and the colonizing microbial biofilms (Chauvat et al., 2011; Chen & Ferris, 1999) and, hence, mostly affect decomposition indirectly by mediating microbial communities. According to the plethora of studies on this topic, the contribution of the fauna to decomposition processes depends on litter traits, environmental conditions, and the taxonomic group studied. Predators impair detritivore activity through top‐down effects, which alters habitat‐specific communities of detritivores and ecosystem processes and can result in trophic cascades (Woodward et al., 2008; Jabiol et al., 2014). As one example, an omnivorous crab predator in an Atlantic U.S. salt marsh counteracted the activity of detritivorous snails on high‐quality detritus, but synergistically interacted with detritivores on low‐quality detritus (Ewers et al., 2012). Kajak (1995) concluded from a literature survey that micro‐predators in soils tend to increase, but macro‐predators tend to decrease, decomposition processes. Hence, the habitat‐specific composition of the litter fauna can be expected to influence whether and how decomposition is affected by animal activities (c.f. Peralta‐Maraver et al., 2019). Exactly how this works, however, often remains obscure. While some detritivore species seem to facilitate each other or interact synergistically (Hedde et al., 2010; Zimmer et al., 2005), others compete for high‐quality food sources (Costantini et al., 2005; Chang et al., 2016) or exert top‐down effects on one another through predation (Ewers et al., 2012). In all these cases, however, their interactions may depend on the quality of their detrital resources. Some studies have suggested that detritivores may occupy a variety of niches, such that niche complementarity allows high detritivore diversity (Wardle, 2006). However, in a saltmarsh system complementarity of detritivores did not explain decomposition process, rather it was the dominant detritivore species that drove decomposition (Treplin et al., 2013). Further, even beyond the borders of biogeographical realms, detritivores exhibit similar preferences for different detrital food sources (Quadros et al., 2015), which suggests that opportunities for niche complementarity are limited. In summary, variation in litter type, and habitat conditions is likely to favor different taxa of detritivores, and these may have species‐specific interactions with each other. In the end, however, the limited opportunities for niche specialization upon litter resources argue that the species composition of the detritivore community may not be a primary factor affecting decomposition rates.

In order to disentangle which factors most greatly affect mass loss rates of tree‐derived leaf litter in coastal habitats, we performed a three‐factor field study with different litter types (oak versus pine) that were incubated in different habitats (upper saltmarsh, creek bank, and coastal forest) in the presence and absence of macrofauna (small‐ versus large‐meshed litterbags). We measured litter fauna and mass loss to test the hypotheses that (1) the fauna composition depends on (1.i) habitat characteristics and (1.ii) litter type; and (2) decomposition rates depend on (2.i) habitat characteristics, (2.ii) litter type, and (2.iii) faunal community composition (mesofauna only versus meso‐ and macrofauna). We predict that the explanatory power of the faunal composition of the detritivore community is lower than that of the litter type; the latter will decrease in relevance over time, while the habitat type will become the most relevant predictor of decomposition processes over time.

## 2 MATERIALS AND METHODS

Our field sites were situated on Sapelo Island, GA, USA (31°27' N, 81°15' W). At this site, the coastal forest includes abundant coastal oak (*Quercus virginiana*) and pine (*Pinus palustris*) trees (Callaway et al., 2002), both henceforth referred to by their common names. Terrestrial, marsh, and freshwater habitats occur in close proximity to each other, and we chose these three habitat types for analyzing the effects of litter quality (oak versus pine), habitat (forest, creek bank, and saltmarsh), and the corresponding meso‐ and macrofauna.

We collected leaf (oak) and needle (pine) litter during spring 2007. According to Zimmer et al. (2002), these litter types represent different qualities of detritus with respect to C:N ratio and condensed tannins (Table 1). Litter was collected in mesh baskets placed below trees; this prevented shed leaves from falling onto the ground, and thereby limited decomposition prior to our study. Litter was returned to the laboratory and air‐dried for >7 days at room temperature. We chose to air‐dry litter to avoid the artifacts that can be caused by oven‐drying. Each litter type was weighed (4.00 ± 0.01 g dry weight) separately into mesh bags. We originally intended to compare litter decomposition in the presence of mesofauna only versus with both meso‐ and macrofauna by enclosing litter in bags with two different mesh sizes (2 × 2 mm² versus 8 × 8 mm²). Bags were placed in groups of four (2 litter types × 2 mesh sizes) with six replicates per habitat (3) and sampling date (4), and embedded in the existing litter present at the site. Since mesh size had only a small effect on the composition of the fauna colonizing the litterbags (see Results), results from small‐ and large‐meshed bags from each habitat*date combination were pooled to describe the faunal assemblages using multidimensional scaling, and we did not explore this contrast further.

**TABLE 1:** Key chemical traits of the different litter types (Zimmer et al., 2002)

	*Quercus virginiana*	*Pinus palustris*
Carbon, mg/g	418 ± 11	447 ± 11
Nitrogen, mg/g	13.2 ± 0.2	10.3 ± 0.5
C*:*N ratio	32	43
Simple phenolics (ferulic acid equivalents), mg/g	15.9 ± 0.5	15.8 ± 0.3
Hydrolyzable tannins (tannic acid equivalents), mg/g	115 ± 3	116 ± 3
Condensed tannins (quebracho equivalents), mg/g	42 ± 9	112 ± 8

Litterbags were deployed in August 2007 and removed from the field after 1 month (September 2007), 6 months (February 2008), 12 months (August 2008), and 25 months (September 2009). Litterbags were individually stored in plastic bags for transport to the University of Georgia Marine Institute (UGAMI), where fauna were extracted in a Berlese apparatus, and the remaining litter cleaned of soil particles, dried at 60°C for 72 hr, and weighed. Invertebrates extracted from the mesh bags were identified to the highest taxonomic level possible and assigned to one of three functional groups: detritivores, predators, or omnivores. For logistical reasons, it was impossible to identify the fauna from litterbags removed from the field in September 2009.

To visualize similarities in the soil fauna between litter types and among habitats, we ran multidimensional scaling with the software package PAST (http://folk.uio.no/ohammer/past/). We analyzed our faunistic data using PERMANOVA and ANOSIM for depicting whether the community composition in the litterbags was driven by the habitat or the litter type. Those taxa that drove differences in community composition were identified through SIMPER analysis. Two‐way ANOVAs served to indicate significant predictors (habitat versus litter type) of litter mass loss over time (GraphPad Prism). To estimate the effects of habitat, litter type, and litter fauna on litter mass loss on each sampling date, we performed regression tree analysis, using the R (http://www.r‐project.org/) package rpart. Regression trees produce predictive models from experimental data by recursively partitioning the data space and developing a prediction model that can be represented graphically as a decision tree (Loh, 2011).

## 3 RESULTS

### 3.1 Detritus‐associated fauna

To test whether the soil fauna composition depends on habitat characteristics or litter type, we distinguished five taxa that we considered entirely detritivorous (including microbivores): Collembola, Gastropoda (*Melampus bidentatus*), Diptera (larvae), Isopoda, and Amphipoda (*Orchestia gryllus*); three taxa that consisted entirely of predators: Arachnida, Pseudoscorpiones, and Chilopoda; and five taxa that included detritivores, predators, or omnivores: Decapoda (*Armases cincereum*), Coleoptera (larvae and adults), Hymenoptera (ants), Acarina, and Nematoda.

The abundance of each taxon varied over time, between habitats, and between litter types (Figure S1). Collembola (springtails) were rare in the marsh where they only occurred in significant numbers after 12 months. Their abundance on oak litter fluctuated over time at the creek bank and in the forest. They were less abundant in the early stages of pine decomposition. Gastropoda (the detritivorous coffee bean snail, *Melampus bidentatus*) were abundant in early litterbags in the marsh but sharply decreased in number over time; they were essentially absent at the creek bank and in the forest. Similarly, Dipteran larvae were abundant during early decomposition, particularly on pine litter, but disappeared over later stages of decomposition. Predators were generally rare and occurred in significant numbers (Arachnida: spiders) only in the forest, mainly on pine litter or during late stages of oak decomposition. Similarly, Coleoptera (beetles) on oak litter were only present in the forest, whereas pine litter‐dwelling beetles were found, albeit in relatively low numbers, during early decomposition in all studied habitats. Acarina (mites) mostly increased in number over the first six months on oak litter, after which their number slightly increased further or remained stable over the following 6 months. On pine litter, this pattern was only observed in the marsh, but they showed a tendency to decrease in numbers initially and then increase again. Less abundant taxa are summarized as "others" in Figure S1 but were considered separately in statistical analyses.

**FIGURE 1:**
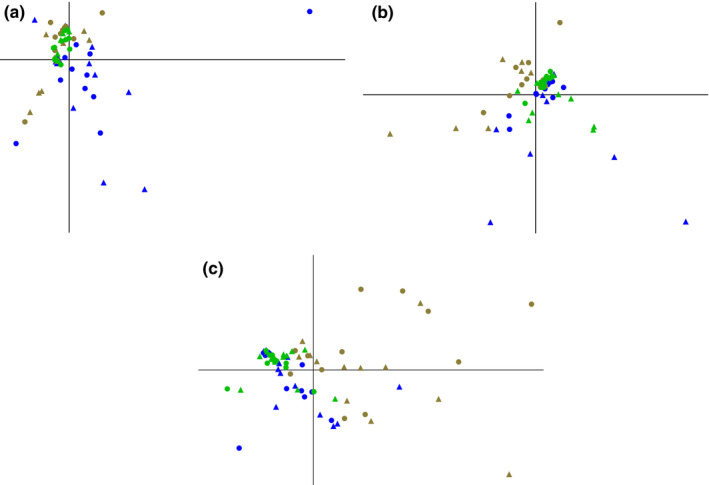
Multidimensional scaling of faunal assemblages in litterbags with respect to different habitats and detrital sources after 1 month (a), 6 months (b), and 12 months (c). Points reflect individual litterbags through their faunistic composition (see also Figure S1 and Table S1). Oak: circles, pine: triangles; marsh: blue; creek bank: green; forest: brown

As indicated by the visualization of faunistic data through nonmetric multidimensional scaling of the faunal data (Figure 1), the dependency of all taxa on different aspects of their environment ("habitat" versus "litter") changed over time. Throughout time, variation among litterbags of the same treatment (litter types and habitats) was high, as indicated by the wide scattering of data points.

After 1 month (Figure 1a), the fauna of the litterbags at the marsh was distinct from that of the other habitats (forest and creek bank) that, in turn, exhibited only very weak distinction. Overall, the individual bags at the creek bank habitat were more similar to each other than those in the marsh or in the forest. After 6 months (Figure 1b), the litterbag fauna of the forest and the marsh were clearly different from each other, while the creek bank fauna was intermediate and exhibited little differences to the marsh fauna. Fauna in litterbags in the creek bank habitat were more different from each other than after one month but still less so than those in the other habitats. A tendency of oak litterbags to cluster together, while pine litterbags became more distinct from each other, became apparent. After 12 months (Figure 1c), the litterbag fauna in the forest differed clearly from that in the marsh but much less so from that at the creek bank. Creek bank and marsh fauna hardly differed from each other and varied less among bags than the forest fauna. These findings were largely corroborated by both PERMANOVA (Table 2), comparing the groups' ("habitat" versus "litter") centroids and their dispersion, and ANOSIM (Table 3), comparing the similarities between and within groups. Both analyses indicated a highly significant effect of "habitat" on the faunal composition of the litterbags. After 6 months, "litter" was also a significant predictor, but "habitat" contributed almost twice as much to the total sum. The most important drivers of faunistic differences among habitats were Acarina (all months), Dipteran larvae (all months), Gastropoda (month 1), and Collembola (month 6 and 12) (SIMPER analysis: Tables S1 and S2; a more detailed analysis of temporal patterns in faunistics in the different habitats and on different litter types using repeated measures ANOVA can be found in Tables S3 and S4).

**TABLE 2:** Two‐way PERMANOVA (9,999 permutations) comparing the faunal composition in litterbags after 1 month (a), 6 months (b), and 12 months (c) as being driven by the experimental factors habitat conditions ("habitat") and litter type ("litter”)

Two‐way PERMANOVA					
Source	SS	*df*	MS	*F*	*p*
(a)					
Habitat	2,443.5	2	1,221.8	5.1405	.0001
Litter	79.1	1	79.1	0.3327	.8170
Interaction	710.1	2	355.0	1.4938	.1736
Residual	15,686.0	66	237.7		
Total	18,919.0	71			
(b)					
Habitat	3,219.1	2	1609.6	4.2755	.0011
Litter	1937.4	1	1937.4	5.1463	.0021
Interaction	767.8	2	383.9	1.0197	.4043
Residual	24,846.0	66	376.5		
Total	30,771.0	71			
(c)					
Habitat	38,124.0	2	19,062.0	17.347	.0001
Litter	1,017.7	1	1,017.7	0.9261	.3802
Interaction	14,477.0	2	738.5	0.6721	.6106
Residual	72,524.0	66	1,098.8		
Total	113,140.0	71			

**TABLE 3:** Two‐way ANOSIM (9,999 permutations) comparing the faunal composition in litterbags after 1 month (a), 6 months (b), and 12 months (c) as being driven by the experimental factors habitat conditions ("habitat") and litter type ("litter)

Two‐way ANOSIM		
Factor	*R*	*p*
(a)		
Habitat	0.15918	.0001
Litter	0.01885	.2281
(b)		
Habitat	0.09229	.0004
Litter	0.08533	.0041
(c)		
Habitat	0.20401	.0001
Litter	−0.02213	.8001

### 3.2 Detrital mass loss

**FIGURE 2:**
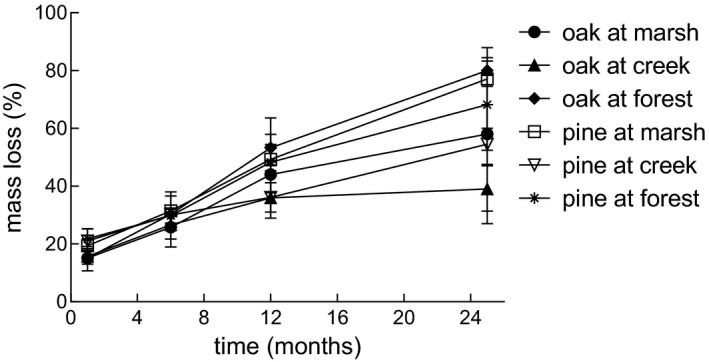
Cumulative mass loss of two litter types (oak versus pine) through the joint action of microbes and fauna over time in three habitats (saltmarsh, creek bank, and forest). Data points are median ± median absolute deviation

Overall, up to ca. 80% of the initial litter mass was lost over 25 months in the marsh and the forest, but only <50% on the creek bank (Figure 2). No clear overall pattern arose with respect to differences in decomposition rates of the different litter types, because the order of litter‐specific decomposition rates changed over time. Individual ANOVAs for each date helped clarify this observation. After both one month (Table 4a) and 6 months (Table 4b) of decomposition, "litter" was the only significant factor (*p* < .001; *p* = .003, respectively) explaining mass loss. Over 12 months (Table 4c), mass loss solely depended on "habitat" (*p* < .001). Over 25 months (Table 4d), the effect of "habitat" (*p* < .001) was significantly shaped by "litter" (habitat x litter interaction: *p* = .007).

**TABLE 4:** ANOVA results, depicting the effects of "habitat" (marsh, creek, forest) and "litter" (oak, pine) on litter mass loss after 1 month (a), 6 months (b), 12 months (c), and 25 months (d)

	SS	*df*	MS	*F* (DFn, DFd)	*p* value
(a) 1 month					
Habitat	9,385	2	4,692	*F* (2, 63) = 2.340	.1047
Litter	53,454	1	53,454	*F* (1, 63) = 26.65	<.0001
Interaction	1,461	2	730.7	*F* (2, 63) = 0.364	.6961
Residual	126,344	63	2005		
(b) 6 months					
Habitat	10,303	2	5,151	*F* (2, 61) = 1.441	.2447
Litter	33,359	1	33,359	*F* (1, 61) = 9.329	.0033
Interaction	9,129	2	4,565	*F* (2, 61) = 1.276	.2864
Residual	218,133	61	3,576		
(c) 12 months					
Habitat	340,948	2	170,474	*F* (2, 64) = 23.42	<.0001
Litter	1,323	1	1,323	*F* (1, 64) = 0.182	.6712
Interaction	32,946	2	16,473	*F* (2, 64) = 2.263	.1123
Residual	465,850	64	7,279		
(d) 25 months					
Habitat	695,594	2	347,797	*F* (2, 38) = 10.52	<.0001
Litter	30,937	1	30,937	*F* (1, 38) = 0.941	.3401
Interaction	369,837	2	184,919	*F* (2, 38) = 5.591	.0073
Residual	1,256,933	38	33,077		

**FIGURE 3:**
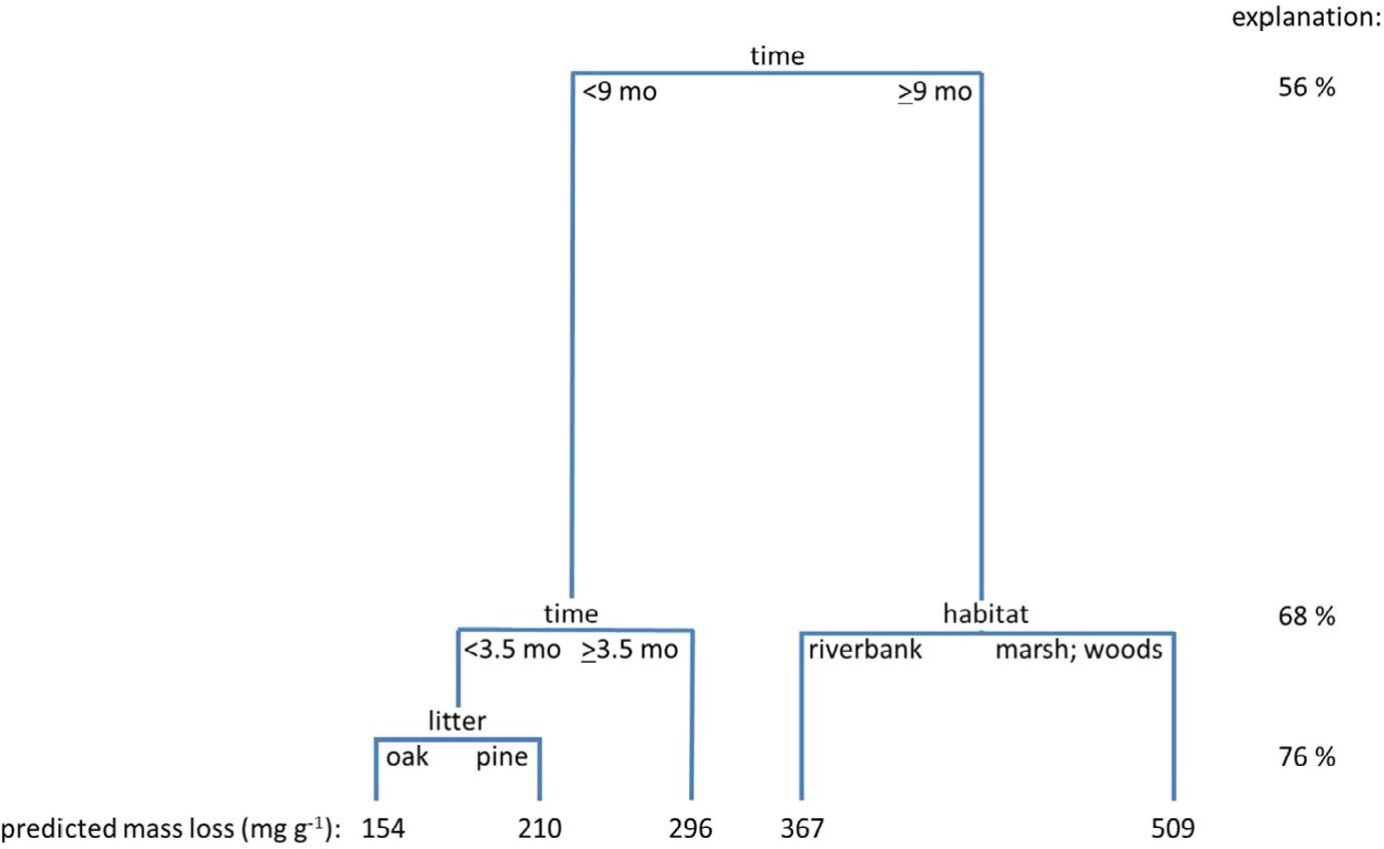
Regression tree explaining 76% of the mass loss of litter as it depends on time, habitat (marsh, creek bank, and forest), and litter type (oak and pine)

According to regression tree analysis (RTA: Figure 3), time was the best predictor of litter mass loss. During the early stages of decomposition (<3.5 months), litter type mediated mass loss rate, with oak predicted to lose 154 mg/g and pine 210 mg/g (76% explained). During later stages (>9 months), habitat type shaped decomposition with lower mass loss rates on the creek bank (predicted: 367 mg/g) than in the saltmarsh or the forest (predicted: 509 mg/g).

**FIGURE 4:**
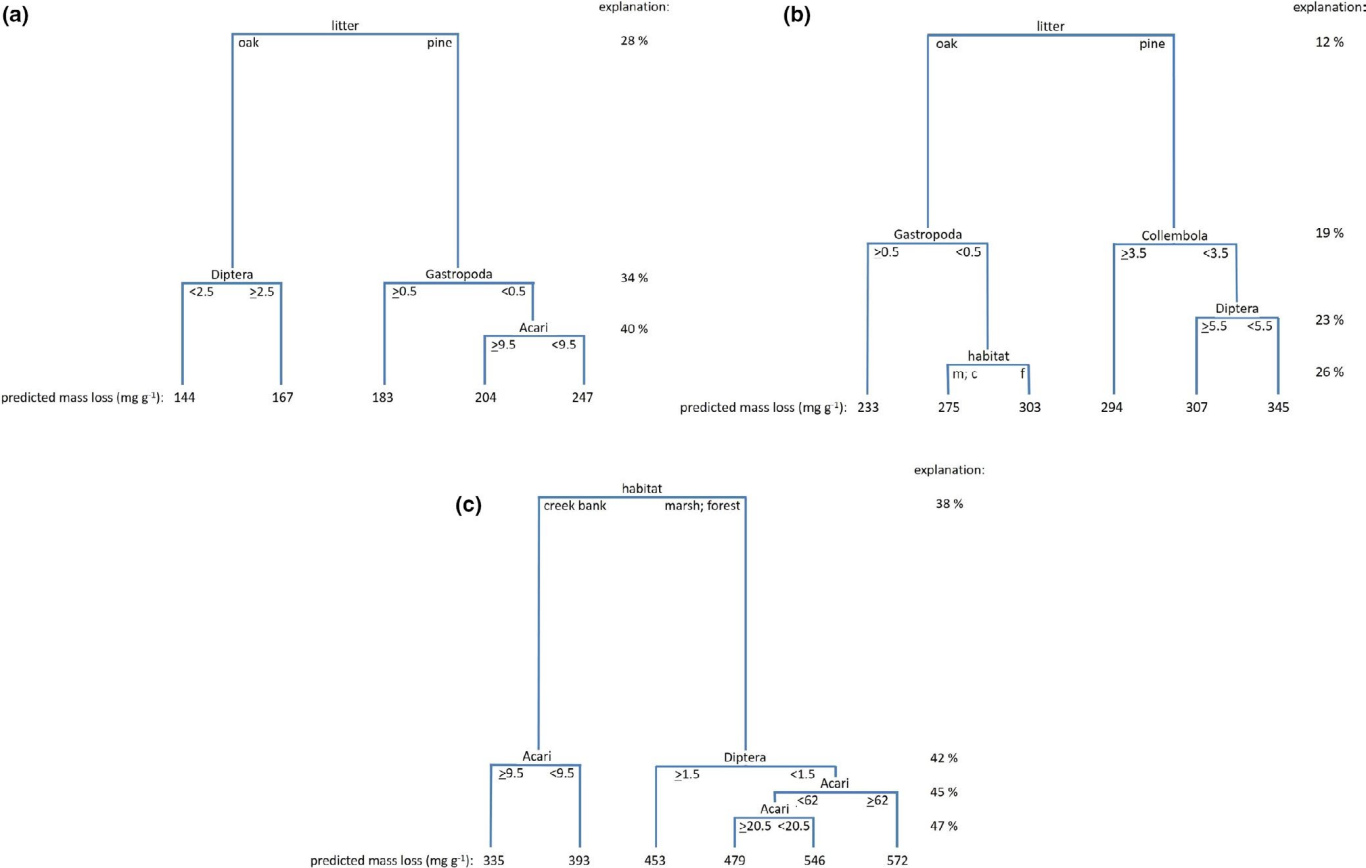
Regression tree explaining the mass loss of different litter types (oak and pine) as it depends on the fauna during 1 month (a), 6 months (b), and 12 months (c)

Decomposition over one month (Figure 4a) was mostly driven by "litter", with pine (predicted: 183–247 mg/g) losing mass faster than oak (predicted: 136–174 mg/g). On oak litter, a higher abundance of Diptera was associated with higher mass loss (predicted: 167 mg/g versus 144 mg/g) (40% explained: Figure 4a). On pine litter, Gastropoda (coffee bean snails) were associated with lower mass loss (predicted: 183 mg/g versus 204–247 mg/g); when there were few coffee bean snails, Acarina (possibly predacious) were also associated with lower mass loss (predicted: 204 mg/g versus 247 mg/g).

Decomposition over 6 months (Figure 4b) was also mediated by "litter", with pine (predicted: 294–354 mg/g) losing mass faster than oak (predicted: 233–303 mg/g) (26% explained: Figure 4b). On oak litter, Gastropoda were associated with low mass loss, and comparing litter with few coffee bean snails, decomposition in the forest was faster (predicted: 303 mg/g) than in the marsh or on the creek bank (predicted: 275 mg/g). On pine litter, Collembola were associated with high mass loss (predicted: 307–345 mg/g versus 294 mg/g), and, when they were abundant, access by Diptera was associated with lower mass loss (predicted: 307 versus 345 mg/g).

In contrast, decomposition over 12 months (Figure 4c) was mostly controlled by "habitat", with higher mass loss in the marsh and forest (predicted: 453–572 mg/g) than on the creek bank (predicted: 335–393 mg/g) (47% explained: Figure 4c). On the creek bank, Acarina (possibly predacious) were associated with lower mass loss (predicted: 335 mg/g versus 393 mg/g). In the forest and marsh, Dipteran larvae were associated with lower mass loss (predicted: 453 mg/g versus 479–572 mg/g). When there were few Dipteran larvae, Acarina were associated with higher mass loss (predicted: 572 mg/g), but when there were few Dipteran larvae and few Acarina (predicted: 479–546 mg/g), mass loss was faster when there were very few Acarina.

## 4 DISCUSSION

**FIGURE 5:**
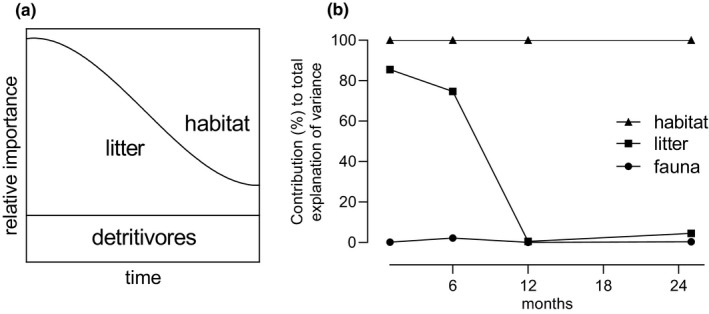
Conceptual model (a) sketching the change in relative importance of the factors species composition of the detritivore fauna, litter type and characteristics, and habitat type over the course of decomposition, as well as confirmation from experimental data (b) based on the cumulative relative contributions of the three factors to explain the total variance of the ANOVA models (Table S5), defining the explained variance of the full model as 100%

Based on the existing knowledge about litter type, faunal composition, and habitat conditions (see Introduction) as drivers of decomposition processes, we developed a conceptual model that describes the change in their relative importance as predictors over time (Figure 5a). Taking into account that even detritivores from different biogeographical realms exhibit similar feeding preferences for contrasting litter types (Quadros et al., 2015), we expected the composition of the detritivore fauna (mesofauna versus meso‐ and macrofauna) to overall exert relatively little effect on the process of decomposition. Although particular taxa of the soil fauna do affect decomposition processes in different ways, these effects might counteract each other in diverse communities. According to Treplin and Zimmer (2012), different leaf litter types become more and more similar over the course of leaching, decay, and decomposition. Hence, we expected the predictive power of the litter type for decomposition processes to decrease over time, while the importance of the habitat conditions would increase at the same time.

Our results confirm that drivers of faunal composition in litter patches (hypothesis 1) and litter decomposition (hypothesis 2; Figure 5b) change over time. Initial colonization of litter patches by the fauna was driven primarily by habitat type, whereas initial detrital mass loss rates were governed primarily by litter traits. However, corroborating previous findings (Treplin & Zimmer, 2012), differences in litter traits diminish upon aging, and over the long term, habitat conditions control both community composition and litter mass loss, confirming both our hypotheses. The mesh size of the litterbags exhibited little effect on the faunal composition and, thus, did not affect litter decomposition as a treatment in our experiment. However, variation in faunal composition within treatments (wide scatter of points in NMDS plots: Figure 1) did exhibit an effect, as shown by RTA (Figures 3 and 4). Larger differences in the species composition of detritivore communities might result in a stronger predictive power of the faunal composition.

Whether it is mostly litter type (hypothesis 1.i) or environmental conditions (habitat: hypothesis 1.ii) that shape the soil animal community has been repeatedly debated. The role of the litter layer in controlling the faunal composition had been one of the major issues in soil ecology since its invention as a biological discipline. Obviously, the presence versus absence of leaf litter shapes the local community composition, as the detrital matter is the basis for detritivore occurrence, and the small‐scale distribution of detritivores depends on litter quality as determined by the decay stage (Ponge, 1999). Corroborating this hypothesis, litter quality affected earthworm (Lumbricidae) communities in a number of mixed deciduous forests (Muys & Lust, 1992; Muys et al., 1992), and converting pure coniferous stands into mixed (deciduous) stands led to marked shifts in springtail (Collembola) communities (Chauvat et al., 2011). However, taking into account that species‐specific differences among leaf litter of different origins diminish over time upon aging, decay, and decomposition of the litter (Treplin & Zimmer, 2012), we hypothesized that litter characteristics would be replaced over time by habitat characteristics as a driving force of litter fauna composition, and the present findings support this hypothesis.

The nature of the abiotic habitat provides constraints on the soil fauna independent of litter type. Soil moisture was a strong driver of (micro‐arthropod) community composition in a coniferous forest in Sweden (Lindberg et al., 2002). Predacious Acarina (Gamasida) density, on the contrary, was controlled by soil type rather than moisture (Wissuwa et al., 2012). Ruf et al. (2003) were able to assign soil fauna assemblages to particular site characteristics. In agricultural landscapes, the soil fauna composition was governed by the habitat, but different animal taxa responded to different habitat characteristics (Dauber et al., 2005; Zimmer et al., 2000). Beyond small‐scale habitat effects on the soil fauna composition, large‐scale habitat characteristics and species‐specific migration patterns among habitat types govern community composition (Martins da Silva et al., 2012). Accordingly, the habitat was the best predictor for the taxonomic composition of the litter fauna in the present study.

Different litter types can vary substantially in traits that affect faunal colonization and decomposition, but these traits are likely to converge with time. In the present study, both observed and predicted mass losses of pine litter were higher than those of oak litter. In contrast to our findings, a geographical comparison of litter mass loss observed little difference between Asian pine and oak species (Sohng et al., 2014). Our results are consistent with past studies on Sapelo Island. In field mesocosms, pine and oak litter exhibited the same mass loss rates when either crab (*Armases cinereum*) or snail (*Melampus bidentatus* and *Litttoraria irrorata*) fed on the litter, but pine litter lost mass significantly faster than oak litter when both crabs and snails were present or when both were absent (Ewers et al., 2012). Similarly, the terrestrial isopod (*Littorophiloscia vittata*) exhibited higher feeding rates on pine litter than oak litter in a laboratory experiment on Sapelo Island (Zimmer et al., 2002).

Species‐specific litter characteristics that drive differences in decomposition rates (e.g., Gessner et al., 2010) diminish quickly upon leaching and early decay, particularly in aquatic environments (c.f. Treplin & Zimmer, 2012). This happens because the most labile compounds are the ones that are most attractive to both microbial decomposers and detritivores—once the leaves are leached and decayed, they mostly consist of recalcitrant and unpalatable structural material, such as cellulose, lignins, and insoluble polyphenolics. Hence, litter mass loss under permanently submerged conditions may be slower in the long run than under terrestrial or tidally affected conditions, as the palatability of the litter decreases more rapidly over time under water than on land.

Our present results corroborate the expectation that the relevance of litter type as driver of both the composition of the litter fauna and litter mass loss diminishes over time, and habitat became the major driver of these processes (Figure 5). As this pattern was observed in all habitats studied herein—terrestrial, freshwater‐influenced, and seawater‐influenced—and was previously shown in terrestrial and aquatic mesocosms (Treplin & Zimmer, 2012), we hypothesize that the diminishing significance of litter characteristics in determining faunal assemblages and litter mass loss over time is a general characteristic of decomposition processes.

Whether or not the abundance and diversity of the (detritivorous) fauna drive decomposition rates may depend on the litter type(s) present (but see Treplin et al., 2013). Thus, the effects of interactions of a diverse fauna are context‐specific: in a laboratory experiment, synergistic interactions of earthworms and isopods promoted the decomposition of high‐ (alder) but not low‐quality (oak) litter (Zimmer et al., 2005). Similarly, millipedes and isopods interacted synergistically and jointly increased decomposition rates under natural rainfall conditions, but this joint effect diminished when rainfall quantity was reduced (Joly et al., 2021). Hence, both habitat conditions and litter quality should be taken into account when predicting decomposition rates based on faunal composition or diversity.

In the present study, the size of the mesh had little effect on the faunal composition or on decomposition rates, especially late in the experiment. This was probably because the macro‐invertebrate taxa that we found in large‐meshed litterbags were also present in many small‐meshed bags, possibly having entered as smaller life stages and developed inside the bags. Hence, it was mostly the surface‐dwelling mega‐fauna (e.g., crabs) that were not present in small‐meshed bags, but this faunal component was also rare in large‐meshed bags. As a result, the fauna found in small‐ and large‐meshed bags differed only slightly, and these differences had little effect on decomposition. This is unlikely to reflect the actual importance of differently sized detritivores, or different detritivore taxa, but simply reflects the challenge in separating the community by size over a long‐term experiment in the field. Because there was considerable variation in faunal composition within treatments, RTA using individual bags as replicates provided more insight into which group of soil fauna was associated with increased or reduced decomposition rates. We speculate that, had our litterbag communities been more different from each other, we might have observed a greater effect of faunal composition on decomposition rates. Further studies should reassess our conclusion that the faunal composition has only little effect. Better approaches in this study system might be to use short‐term field incubations to isolate the importance of different‐sized individuals using different meshes, or to use mesocosm experiments to isolate the importance of different taxa.

Regression tree analysis indicated that some of the taxa in our samples (Gastropoda and Acarina) were associated with low litter mass loss rates, whereas we found contrasting associations for others (Dipteran larvae, Collembola, and Acarina in combination). These taxa were also those that drove differences in faunal composition across habitats, according to SIMPER analyses. As RTA, however, is purely correlative and not causative, our interpretation of these findings remains inevitably speculative and warrants further testing. Gastropoda in our litterbags were exclusively coffee bean snails (*Melampus bidentatus*), a common species in saltmarshes along the entire U.S. East coast (Lee & Silliman, 2006). The mucus that snails leave on detrital matter promotes microbial activity (Theenhaus & Scheu, 1996; c.f. Zimmer et al., 2005) and would, hence, be expected to improve litter mass loss. It is possible, however, that this positive effect is counteracted by reduced palatability of detrital matter with snail mucus to other detritivores, or by grazing of snails upon microbial films.

Acarina are a trophically very diverse group. Reduction in litter mass loss through the presence of this mesofaunal taxon may be caused by most of the species found in litterbags in this study being predacious (potentially including microbivorous) rather than detritivorous. According to Kajak (1995), micro‐predators (e.g., Acarina) in soils tend to increase but macro‐predators tend to decrease decomposition processes. This is in contrast to our findings, but any conclusive interpretation of our results would require detailed studies of how the various animals interact with each other and with the microbial communities.

Dipteran larvae and Collembola are commonly considered detritivorous. Hence, the decreased litter mass loss that we observed in the presence of these taxa is in contrast with a large body of literature (for review: Kamplicher & Bruckner, 2009). Again, understanding this finding will require more detailed studies of species interactions among the soil fauna (see also: Eisenhauer et al., 2017).

We note that, although regression tree analysis did identify some faunal effects on decomposition, the roles of these species were always small and secondary to the primary factor (either litter type or habitat) affecting decomposition on each date. Again, this conclusion might have been different if we had been able to create larger differences in faunal composition, and we agree that the details of the interactions among detritivore species and their effects on decomposition are still ripe areas for further investigation.

We conclude that, although the habitat type is the principle driver of the faunal community composition of the litter layer, litter quality is a significant driver of litter mass loss in the early stages of the decomposition process. With time, however, litter types become more and more similar (chemically and structurally: Treplin & Zimmer, 2012) so that habitat becomes the dominating factor in determining both fauna and decomposition processes when litter ages. Hence, how the litter of terrestrial trees growing in coastal areas is decomposed depends primarily on where it is transported upon leaf fall—to terrestrial, freshwater, or marine habitats. More generally, considering how the factors that affect decomposition change over time may help reconcile seemingly disparate findings in the literature.

## CONFLICT OF INTEREST

The authors declare no conflict of interest.

## AUTHOR CONTRIBUTION

**Franziska K. Seer:** Conceptualization (supporting); Funding acquisition (equal); Investigation (equal); Writing‐review & editing (supporting). **Gregor Putze:** Conceptualization (supporting); Funding acquisition (equal); Investigation (equal); Writing‐review & editing (supporting). **Steven C. Pennings:** Conceptualization (equal); Investigation (equal); Methodology (equal); Supervision (equal); Validation (equal); Writing‐review & editing (equal). **Martin Zimmer:** Conceptualization (equal); Methodology (equal); Resources (equal); Supervision (lead); Validation (equal); Visualization (lead); Writing‐original draft (lead); Writing‐review & editing (equal).

## DATA AVAILABILITY STATEMENT

The data have been deposited with PANGAEA: https://doi.org/10.1594/PANGAEA.931664

## Supporting information

Appendix S1

Table S1

Table S2

Table S3

Table S4

Table S5
