# Supplementary material for: Drivers of litter mass loss and faunal composition of detritus patches change over time
Source: Ecol Evol. 2021 Jun 23;11(14):9642–51. doi: 10.1002/ece3.7787 (PMC8293728; doi:10.1002/ece3.7787)
Supplement: Supplementary file 1 — Appendix S1 [file ECE3-11-9642-s005.docx]

Supplementary Figure 1: Changes in faunal composition (% of total abundance) in oak *versus* pine litter bags in different habitats over time. Shadings distinguish taxonomic groups (see legend); colors distinguish feeding types – brown: detritivores, red: carnivores, orange: omnivores, blue: taxa with diverse feeding types; numbers above the bars indicate the total number of individuals captured.

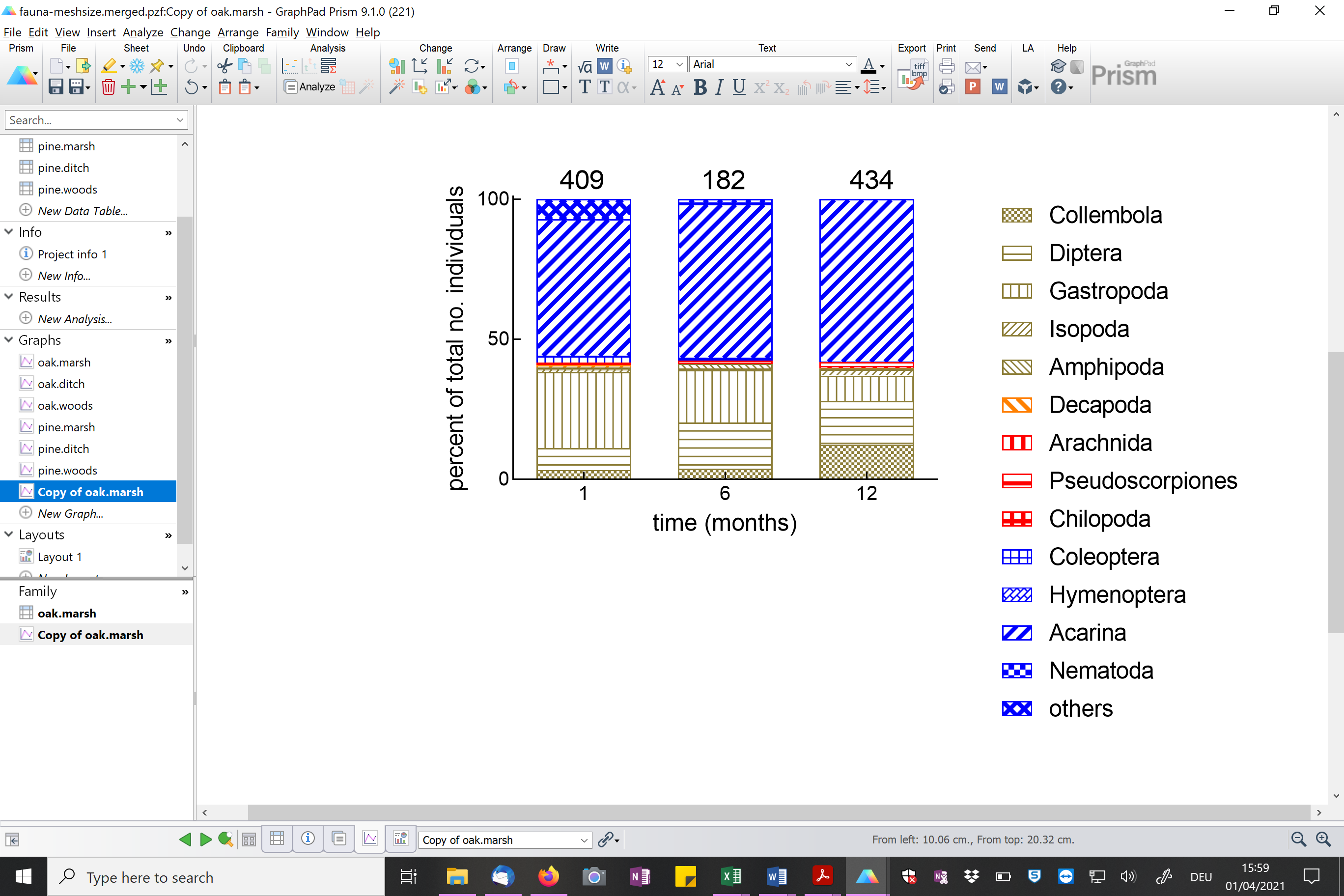


Supplementary Table 1: Repeated-measures ANOVA results, explaining changes in the dependency of faunal abundances on the environmental factors "habitat" and "litter" over "time".

Supplementary Table 2: ANOVA results for different time periods, explaining the dependency of different faunal taxa on the environmental parameters "habitat" and "litter" after 1 month (A), 6 months (B), and 12 months (C).

Supplementary Table 3: ANOVA results, explaining the effects of "habitat" (marsh, creek, woods), "litter" (oak, pine) and "fauna" presence of mesofauna versus mesofauna + macrofauna) on litter mass loss after 1 month (A), 6 months (B), 12 months (C), and 25 months (D).
