# Supplementary material for: Drivers of litter mass loss and faunal composition of detritus patches change over time
Source: Ecol Evol. 2021 Jun 23;11(14):9642–51. doi: 10.1002/ece3.7787 (PMC8293728; doi:10.1002/ece3.7787)
Supplement: Supplementary file 2 — Table S1 [file ECE3-11-9642-s003.docx]

Supplementary Table 1: Results of SIMPER-analyses on taxon-specific contributions to faunistic differences among habitats(i: marsh vs. creek bank; ii: marsh vs. forest; iii: creek bank vs. forest) after 1 month (A), 6 months (B), and 12 months (C).

| **A** |  |  |  |  |  |
| --- | --- | --- | --- | --- | --- |
| **i** | **Av. Dissim.** | **Contrib. %** | **Cumulative %** | **Mean marsh** | **Mean creek** |
| Gastropoda | 22.8 | 35.3 | 35.3 | 11.4 | 0.5 |
| Acarina | 20.1 | 31.1 | 66.4 | 12.8 | 7.7 |
| Diptera | 10.2 | 15.7 | 82.1 | 4.8 | 2.2 |
| Collembola | 5.7 | 8.8 | 91.0 | 1.5 | 1.2 |
| Coleoptera | 2.5 | 3.9 | 94.9 | 1.0 | 0.5 |
| Isopoda | 0.8 | 1.2 | 96.1 | 0.3 | 0.0 |
| Arachnida | 0.6 | 0.9 | 96.9 | 0.2 | 0.0 |
| Amphipoda | 0.5 | 0.8 | 97.7 | 0.2 | 0.0 |
| Decapoda | 0.4 | 0.6 | 98.3 | 0.2 | 0.0 |
| Pseudoscorpiones | 0.4 | 0.6 | 98.9 | 0.2 | 0.0 |
| Nematoda | 0.3 | 0.4 | 99.3 | 0.0 | 0.1 |
| Chilopoda | 0.2 | 0.4 | 99.7 | 0.0 | 0.1 |
| Hymenoptera | 0.2 | 0.3 | 100.0 | 0.1 | 0.0 |
|  |  |  |  |  |  |
| **ii** | **Av. Dissim.** | **Contrib. %** | **Cumulative %** | **Mean marsh** | **Mean woods** |
| Acarina | 21.5 | 31.4 | 31.4 | 12.8 | 9.0 |
| Gastropoda | 21.2 | 30.9 | 62.3 | 11.4 | 0.1 |
| Diptera | 11.4 | 16.6 | 78.9 | 4.8 | 4.5 |
| Collembola | 7.4 | 10.9 | 89.8 | 1.5 | 2.6 |
| Coleoptera | 2.9 | 4.2 | 94.0 | 1.0 | 1.0 |
| Isopoda | 1.2 | 1.7 | 95.7 | 0.3 | 0.3 |
| Arachnida | 0.9 | 1.3 | 97.0 | 0.2 | 0.3 |
| Pseudoscorpiones | 0.7 | 1.0 | 98.1 | 0.2 | 0.2 |
| Hymenoptera | 0.5 | 0.8 | 98.8 | 0.1 | 0.2 |
| Amphipoda | 0.4 | 0.6 | 99.5 | 0.2 | 0.0 |
| Decapoda | 0.4 | 0.5 | 100.0 | 0.2 | 0.0 |
| Chilopoda | 0.0 | 0.0 | 100.0 | 0.0 | 0.0 |
| Nematoda | 0.0 | 0.0 | 100.0 | 0.0 | 0.0 |
|  |  |  |  |  |  |
| **iii** | **Av. Dissim.** | **Contrib. %** | **Cumulative %** | **Mean creek** | **Mean woods** |
| Acarina | 24.8 | 43.4 | 43.4 | 7.7 | 9.0 |
| Diptera | 12.8 | 22.5 | 65.9 | 2.2 | 4.5 |
| Collembola | 9.3 | 16.3 | 82.1 | 1.2 | 2.6 |
| Coleoptera | 3.6 | 6.3 | 88.4 | 0.5 | 1.0 |
| Gastropoda | 2.2 | 3.8 | 92.3 | 0.5 | 0.1 |
| Isopoda | 1.1 | 2.0 | 94.2 | 0.0 | 0.3 |
| Arachnida | 1.0 | 1.7 | 95.9 | 0.0 | 0.3 |
| Pseudoscorpiones | 0.8 | 1.4 | 97.3 | 0.0 | 0.2 |
| Hymenoptera | 0.5 | 0.9 | 98.2 | 0.0 | 0.2 |
| Nematoda | 0.4 | 0.7 | 98.9 | 0.1 | 0.0 |
| Amphipoda | 0.3 | 0.6 | 99.4 | 0.0 | 0.0 |
| Chilopoda | 0.3 | 0.6 | 100.0 | 0.1 | 0.0 |
| Decapoda | 0.0 | 0.0 | 100.0 | 0.0 | 0.0 |

| **B** |  |  |  |  |  |
| --- | --- | --- | --- | --- | --- |
| **i** | **Av. Dissim.** | **Contrib. %** | **Cumulative %** | **Mean marsh** | **Mean creek** |
| Acarina | 29.0 | 39.9 | 39.9 | 11.0 | 5.5 |
| Diptera | 21.5 | 29.6 | 69.6 | 8.6 | 5.1 |
| Gastropoda | 10.5 | 14.5 | 84.0 | 4.3 | 0.3 |
| Collembola | 8.0 | 11.0 | 95.0 | 0.5 | 2.1 |
| Amphipoda | 1.1 | 1.5 | 96.5 | 0.2 | 0.0 |
| Nematoda | 0.9 | 1.2 | 97.8 | 0.0 | 0.2 |
| Isopoda | 0.6 | 0.8 | 98.5 | 0.2 | 0.0 |
| Pseudoscorpiones | 0.4 | 0.6 | 99.1 | 0.0 | 0.0 |
| Arachnida | 0.3 | 0.4 | 99.5 | 0.0 | 0.0 |
| Hymenoptera | 0.2 | 0.3 | 99.7 | 0.0 | 0.0 |
| Coleoptera | 0.1 | 0.2 | 99.9 | 0.0 | 0.0 |
| Decaopoda | 0.1 | 0.1 | 100.0 | 0.0 | 0.0 |
| Chilopoda | 0.0 | 0.0 | 100.0 | 0.0 | 0.0 |
|  |  |  |  |  |  |
| **ii** | **Av. Dissim.** | **Contrib. %** | **Cumulative %** | **Mean marsh** | **Mean woods** |
| Acarina | 28.4 | 38.8 | 38.8 | 11.0 | 16.1 |
| Collembola | 17.7 | 24.1 | 62.9 | 0.5 | 7.8 |
| Diptera | 12.0 | 16.4 | 79.2 | 8.6 | 0.4 |
| Gastropoda | 8.0 | 10.9 | 90.1 | 4.3 | 0.1 |
| Arachnida | 3.1 | 4.2 | 94.3 | 0.0 | 1.5 |
| Nematoda | 1.1 | 1.5 | 95.8 | 0.0 | 0.2 |
| Pseudoscorpiones | 0.7 | 0.9 | 96.7 | 0.0 | 0.2 |
| Coleoptera | 0.6 | 0.9 | 97.5 | 0.0 | 0.3 |
| Chilopoda | 0.6 | 0.9 | 98.4 | 0.0 | 0.2 |
| Amphipoda | 0.5 | 0.7 | 99.1 | 0.2 | 0.0 |
| Isopoda | 0.4 | 0.6 | 99.7 | 0.2 | 0.0 |
| Hymenoptera | 0.1 | 0.2 | 99.9 | 0.0 | 0.0 |
| Decapoda | 0.1 | 0.1 | 100.0 | 0.0 | 0.0 |
|  |  |  |  |  |  |
| **iii** | **Av. Dissim.** | **Contrib. %** | **Cumulative %** | **Mean creek** | **Mean woods** |
| Acarina | 27.4 | 41.1 | 41.1 | 5.5 | 16.1 |
| Collembola | 18.7 | 28.1 | 69.2 | 2.1 | 7.8 |
| Diptera | 11.8 | 17.8 | 86.9 | 5.1 | 0.4 |
| Arachnida | 3.3 | 5.0 | 91.9 | 0.0 | 1.5 |
| Nematoda | 1.7 | 2.5 | 94.5 | 0.2 | 0.2 |
| Gastropoda | 1.5 | 2.3 | 96.8 | 0.3 | 0.1 |
| Chilopoda | 0.7 | 1.1 | 97.9 | 0.0 | 0.2 |
| Coleoptera | 0.6 | 1.0 | 98.8 | 0.0 | 0.3 |
| Pseudoscorpiones | 0.5 | 0.8 | 99.6 | 0.0 | 0.2 |
| Amphipoda | 0.3 | 0.4 | 100.0 | 0.0 | 0.0 |
| Isopoda | 0.0 | 0.0 | 100.0 | 0.0 | 0.0 |
| Decapoda | 0.0 | 0.0 | 100.0 | 0.0 | 0.0 |
| Hymenoptera | 0.0 | 0.0 | 100.0 | 0.0 | 0.0 |

| **C** |  |  |  |  |  |
| --- | --- | --- | --- | --- | --- |
| **i** | **Av. Dissim.** | **Contrib. %** | **Cumulative %** | **Mean marsh** | **Mean creek** |
| Acarina | 32.8 | 50.5 | 50.5 | 25.0 | 12.0 |
| Collembola | 12.3 | 18.9 | 69.5 | 6.1 | 6.8 |
| Diptera | 11.0 | 17.0 | 86.5 | 4.1 | 5.0 |
| Gastropoda | 3.7 | 5.7 | 92.2 | 3.0 | 0.0 |
| Isopoda | 2.3 | 3.6 | 95.8 | 2.1 | 0.0 |
| Pseudoscorpiones | 0.7 | 1.1 | 96.9 | 0.5 | 0.0 |
| Arachnida | 0.7 | 1.1 | 97.9 | 0.2 | 0.2 |
| Coleoptera | 0.5 | 0.8 | 98.7 | 0.1 | 0.3 |
| Hymenoptera | 0.3 | 0.5 | 99.2 | 0.1 | 0.1 |
| Amphipoda | 0.3 | 0.5 | 99.7 | 0.1 | 0.0 |
| Decapoda | 0.2 | 0.4 | 100.0 | 0.0 | 0.0 |
| Chilopoda | 0.0 | 0.0 | 100.0 | 0.0 | 0.0 |
| Nematoda | 0.0 | 0.0 | 100.0 | 0.0 | 0.0 |
|  |  |  |  |  |  |
| **ii** | **Av. Dissim.** | **Contrib. %** | **Cumulative %** | **Mean marsh** | **Mean woods** |
| Acarina | 32.8 | 50.5 | 50.5 | 25.0 | 12.0 |
| Collembola | 12.3 | 18.9 | 69.5 | 6.1 | 6.8 |
| Diptera | 11.0 | 17.0 | 86.5 | 4.1 | 5.0 |
| Gastropoda | 3.7 | 5.7 | 92.2 | 3.0 | 0.0 |
| Isopoda | 2.3 | 3.6 | 95.8 | 2.1 | 0.0 |
| Pseudoscorpiones | 0.7 | 1.1 | 96.9 | 0.5 | 0.0 |
| Arachnida | 0.7 | 1.1 | 97.9 | 0.2 | 0.2 |
| Coleoptera | 0.5 | 0.8 | 98.7 | 0.1 | 0.3 |
| Hymenoptera | 0.3 | 0.5 | 99.2 | 0.1 | 0.1 |
| Amphipoda | 0.3 | 0.5 | 99.7 | 0.1 | 0.0 |
| Decapoda | 0.2 | 0.4 | 100.0 | 0.0 | 0.0 |
| Chilopoda | 0.0 | 0.0 | 100.0 | 0.0 | 0.0 |
| Nematoda | 0.0 | 0.0 | 100.0 | 0.0 | 0.0 |
|  |  |  |  |  |  |
| **iii** | **Av. Dissim.** | **Contrib. %** | **Cumulative %** | **Mean creek** | **Mean woods** |
| Acarina | 30.6 | 45.0 | 45.0 | 12.0 | 49.0 |
| Collembola | 28.1 | 41.4 | 86.4 | 6.8 | 42.4 |
| Diptera | 4.3 | 6.3 | 92.7 | 5.0 | 0.5 |
| Arachnida | 1.2 | 1.7 | 94.4 | 0.2 | 1.3 |
| Chilopoda | 0.9 | 1.4 | 95.8 | 0.0 | 0.9 |
| Coleoptera | 0.7 | 1.0 | 96.8 | 0.3 | 0.6 |
| Pseudoscorpiones | 0.7 | 1.0 | 97.8 | 0.0 | 0.6 |
| Hymenoptera | 0.6 | 0.9 | 98.7 | 0.1 | 0.7 |
| Isopoda | 0.5 | 0.7 | 99.4 | 0.0 | 0.6 |
| Nematoda | 0.3 | 0.5 | 99.8 | 0.0 | 0.5 |
| Gastropoda | 0.1 | 0.2 | 100.0 | 0.0 | 0.1 |
| Amphipoda | 0.0 | 0.0 | 100.0 | 0.0 | 0.0 |
| Decapoda | 0.0 | 0.0 | 100.0 | 0.0 | 0.0 |
