# Supplementary material for: Drivers of litter mass loss and faunal composition of detritus patches change over time
Source: Ecol Evol. 2021 Jun 23;11(14):9642–51. doi: 10.1002/ece3.7787 (PMC8293728; doi:10.1002/ece3.7787)
Supplement: Supplementary file 3 — Table S2 [file ECE3-11-9642-s002.docx]

Supplementary Table 2: Results of SIMPER-analyses on taxon-specific contributions to faunistic differences across all habitats after 1 month (A), 6 months (B), and 12 months (C).

| **A** | **Av. Dissim.** | **Contrib. %** | **Cumulative %** | **Mean marsh** | **Mean creek** | **Mean woods** |
| --- | --- | --- | --- | --- | --- | --- |
| Acariina | 22.1 | 34.9 | 34.9 | 12.8 | 7.7 | 9.0 |
| Gastropoda | 15.4 | 24.3 | 59.2 | 11.4 | 0.5 | 0.1 |
| Diptera | 11.5 | 18.1 | 77.3 | 4.8 | 2.2 | 4.5 |
| Collembola | 7.5 | 11.8 | 89.0 | 1.5 | 1.2 | 2.6 |
| Coleoptera | 3.0 | 4.7 | 93.8 | 1.0 | 0.5 | 1.0 |
| Isopoda | 1.0 | 1.6 | 95.4 | 0.3 | 0.0 | 0.3 |
| Arachnida | 0.8 | 1.3 | 96.7 | 0.2 | 0.0 | 0.3 |
| Pseudoscorpiones | 0.6 | 1.0 | 97.6 | 0.2 | 0.0 | 0.2 |
| Hymenoptera | 0.4 | 0.7 | 98.3 | 0.1 | 0.0 | 0.2 |
| Amphipoda | 0.4 | 0.7 | 99.0 | 0.2 | 0.0 | 0.0 |
| Decapoda | 0.3 | 0.4 | 99.4 | 0.2 | 0.0 | 0.0 |
| Nematoda | 0.2 | 0.3 | 99.7 | 0.0 | 0.1 | 0.0 |
| Chilopoda | 0.2 | 0.3 | 100.0 | 0.0 | 0.1 | 0.0 |

| **B** | **Av. Dissim.** | **Contrib. %** | **Cumulative %** | **Mean marsh** | **Mean creek** | **Mean woods** |
| --- | --- | --- | --- | --- | --- | --- |
| Acarina | 28.2 | 39.9 | 39.9 | 11.0 | 5.5 | 16.1 |
| Diptera | 15.0 | 21.2 | 61.1 | 8.6 | 5.1 | 0.4 |
| Collembola | 14.9 | 21.1 | 82.2 | 0.5 | 2.1 | 7.8 |
| Gastropoda | 6.5 | 9.3 | 91.4 | 4.3 | 0.3 | 0.1 |
| Arachnida | 2.3 | 3.2 | 94.6 | 0.0 | 0.0 | 1.5 |
| Nematoda | 1.2 | 1.7 | 96.4 | 0.0 | 0.2 | 0.2 |
| Amphipoda | 0.6 | 0.9 | 97.2 | 0.2 | 0.0 | 0.0 |
| Pseudoscorpiones | 0.5 | 0.8 | 98.0 | 0.0 | 0.0 | 0.2 |
| Coleoptera | 0.5 | 0.7 | 98.7 | 0.0 | 0.0 | 0.3 |
| Chilopoda | 0.5 | 0.7 | 99.3 | 0.0 | 0.0 | 0.2 |
| Isopoda | 0.3 | 0.5 | 99.8 | 0.2 | 0.0 | 0.0 |
| Hymenoptera | 0.1 | 0.2 | 99.9 | 0.0 | 0.0 | 0.0 |
| Decapoda | 0.0 | 0.1 | 100.0 | 0.0 | 0.0 | 0.0 |

| **C** | **Av. Dissim.** | **Contrib. %** | **Cumulative %** | **Mean marsh** | **Mean creek** | **Mean woods** |
| --- | --- | --- | --- | --- | --- | --- |
| Acarina | 30.0 | 45.6 | 45.6 | 25.0 | 12.0 | 49.0 |
| Collembola | 22.6 | 34.3 | 79.9 | 6.1 | 6.8 | 42.4 |
| Diptera | 6.0 | 9.1 | 89.0 | 4.1 | 5.0 | 0.5 |
| Gastropoda | 2.0 | 3.0 | 92.0 | 3.0 | 0.0 | 0.1 |
| Isopoda | 1.5 | 2.3 | 94.3 | 2.1 | 0.0 | 0.6 |
| Arachnida | 1.0 | 1.5 | 95.8 | 0.2 | 0.2 | 1.3 |
| Pseudoscorpiones | 0.7 | 1.1 | 96.8 | 0.5 | 0.0 | 0.6 |
| Chilopoda | 0.6 | 0.9 | 97.7 | 0.0 | 0.0 | 0.9 |
| Coleoptera | 0.6 | 0.9 | 98.6 | 0.1 | 0.3 | 0.6 |
| Hymenoptera | 0.5 | 0.8 | 99.4 | 0.1 | 0.1 | 0.7 |
| Nematoda | 0.2 | 0.3 | 99.7 | 0.0 | 0.0 | 0.5 |
| Amphipoda | 0.1 | 0.2 | 99.9 | 0.1 | 0.0 | 0.0 |
| Decapoda | 0.1 | 0.1 | 100.0 | 0.0 | 0.0 | 0.0 |
