# Supplementary material for: Drivers of litter mass loss and faunal composition of detritus patches change over time
Source: Ecol Evol. 2021 Jun 23;11(14):9642–51. doi: 10.1002/ece3.7787 (PMC8293728; doi:10.1002/ece3.7787)
Supplement: Supplementary file 4 — Table S3 [file ECE3-11-9642-s004.docx]

Supplementary Table 3: Repeated-measures ANOVA results, explaining changes in the dependency of faunal abundances on the environmental factors "habitat" and "litter" over "time".

|  | | df | F | p |  |  | | df | F | p |
| --- | --- | --- | --- | --- | --- | --- | --- | --- | --- | --- |
| time | Acarina | 2 | 30.39 | <0.001 |  | habitat | Acarina | 2 | 10.57 | <0.001 |
|  | Amphipoda  (*Orchestia gryllus*) | 2 | 0.79 | >0.378 |  |  | Amphipoda  (*Orchestia gryllus*) | 2 | 5.46 | 0.006 |
|  | Arachnida | 2 | 0.83 | >0.365 |  |  | Arachnida | 2 | 6.90 | 0.002 |
|  | Chilopoda | 2 | 4.47 | <0.038 |  |  | Chilopoda | 2 | 8.53 | 0.001 |
|  | Coleoptera | 2 | 12.25 | <0.001 |  |  | Coleoptera | 2 | 3.42 | 0.038 |
|  | Collembola | 2 | 44.47 | <0.001 |  |  | Collembola | 2 | 40.99 | <0.001 |
|  | Decapoda  (*Armases cinereum*) | 2 | 0.73 | >0.397 |  |  | Decapoda  (*Armases cinereum*) | 2 | 3.19 | 0.047 |
|  | Dipteran larvae | 2 | 0.52 | >0.457 |  |  | Dipteran larvae | 2 | 3.44 | 0.038 |
|  | Gastropoda  (*Melampus bidentatus*) | 2 | 16.44 | <0.001 |  |  | Gastropoda  (*Melampus bidentatus*) | 2 | 16.30 | <0.001 |
|  | Hymenoptera | 2 | 7.49 | <0.008 |  |  | Hymenoptera | 2 | 4.32 | 0.017 |
|  | Isopoda | 2 | 3.53 | <0.065 |  |  | Isopoda | 2 | 3.54 | 0.035 |
|  | Nematoda | 2 | 0.86 | >0.358 |  |  | Nematoda | 2 | 1.94 | 0.152 |
|  | Pseudoscorpiones | 2 | 6.42 | <0.014 |  |  | Pseudoscorpiones | 2 | 5.99 | 0.004 |
|  | "Others" | 2 | 20.69 | <0.001 |  |  | "Others" | 2 | 2.60 | 0.082 |
| time X habitat | Acarina | 4 | 8.58 | <0.001 |  | litter | Acarina | 1 | 2.51 | 0.118 |
|  | Amphipoda  (*Orchestia gryllus*) | 4 | 0.32 | >0.731 |  |  | Amphipoda  (*Orchestia gryllus*) | 1 | 0.66 | 0.421 |
|  | Arachnida | 4 | 1.07 | >0.350 |  |  | Arachnida | 1 | 0.28 | 0.599 |
|  | Chilopoda | 4 | 5.58 | <0.006 |  |  | Chilopoda | 1 | 1.36 | 0.248 |
|  | Coleoptera | 4 | 1.13 | >0.328 |  |  | Coleoptera | 1 | 4.84 | 0.031 |
|  | Collembola | 4 | 20.52 | <0.001 |  |  | Collembola | 1 | 0.02 | 0.885 |
|  | Decapoda  (*Armases cinereum*) | 4 | 0.73 | >0.487 |  |  | Decapoda  (*Armases cinereum*) | 1 | 0.36 | 0.553 |
|  | Dipteran larvae | 4 | 2.02 | >0.096 |  |  | Dipteran larvae | 1 | 4.42 | 0.039 |
|  | Gastropoda  (*Melampus bidentatus*) | 4 | 14.41 | <0.001 |  |  | Gastropoda  (*Melampus bidentatus*) | 1 | 0.41 | 0.526 |
|  | Hymenoptera | 4 | 3.85 | <0.026 |  |  | Hymenoptera | 1 | 0.36 | 0.551 |
|  | Isopoda | 4 | 1.88 | >0.118 |  |  | Isopoda | 1 | 1.63 | 0.207 |
|  | Nematoda | 4 | 1.14 | >0.327 |  |  | Nematoda | 1 | 0.80 | 0.347 |
|  | Pseudoscorpiones | 4 | 1.20 | >0.306 |  |  | Pseudoscorpiones | 1 | 3.10 | 0.083 |
|  | "Others" | 4 | 3.19 | <0.048 |  |  | "Others" | 1 | 1.31 | 0.257 |
| time X litter | Acarina | 2 | 1.43 | >0.236 |  | habitat X litter | Acarina | 2 | 0.69 | 0.506 |
|  | Amphipoda  (*Orchestia gryllus*) | 2 | 0.03 | >0.860 |  |  | Amphipoda  (*Orchestia gryllus*) | 2 | 0.29 | 0.751 |
|  | Arachnida | 2 | 0.80 | >0.374 |  |  | Arachnida | 2 | 0.23 | 0.794 |
|  | Chilopoda | 2 | 1.90 | >0.173 |  |  | Chilopoda | 2 | 1.36 | 0.264 |
|  | Coleoptera | 2 | 1.17 | >0.284 |  |  | Coleoptera | 2 | 0.08 | 0.924 |
|  | Collembola | 2 | 0.55 | >0.460 |  |  | Collembola | 2 | 1.11 | 0.334 |
|  | Decapoda  (*Armases cinereum*) | 2 | 0.57 | >0.454 |  |  | Decapoda  (*Armases cinereum*) | 2 | 0.36 | 0.703 |
|  | Dipteran larvae | 2 | 5.04 | <0.028 |  |  | Dipteran larvae | 2 | 0.63 | 0.533 |
|  | Gastropoda  (*Melampus bidentatus*) | 2 | 0.95 | >0.333 |  |  | Gastropoda  (*Melampus bidentatus*) | 2 | 0.56 | 0.572 |
|  | Hymenoptera | 2 | 0.39 | >0.532 |  |  | Hymenoptera | 2 | 0.36 | 0.700 |
|  | Isopoda | 2 | 1.24 | >0.270 |  |  | Isopoda | 2 | 1.26 | 0.290 |
|  | Nematoda | 2 | 0.37 | >0.547 |  |  | Nematoda | 2 | 0.36 | 0.702 |
|  | Pseudoscorpiones | 2 | 0.64 | >0.428 |  |  | Pseudoscorpiones | 2 | 2.58 | 0.084 |
|  | "Others" | 2 | 2.28 | >0.136 |  |  | "Others" | 2 | 1.22 | 0.300 |
| time X habitat X litter | Acarina | 4 | 0.37 | >0.691 |  |  |  |  |  |  |
|  | Amphipoda  (*Orchestia gryllus*) | 4 | 0.32 | >0.731 |  |  |  |  |  |  |
|  | Arachnida | 4 | 0.63 | >0.535 |  |  |  |  |  |  |
|  | Chilopoda | 4 | 1.90 | >0.158 |  |  |  |  |  |  |
|  | Coleoptera | 4 | 0.14 | >0.870 |  |  |  |  |  |  |
|  | Collembola | 4 | 1.99 | >0.100 |  |  |  |  |  |  |
|  | Decapoda  (*Armases cinereum*) | 4 | 0.57 | >0.570 |  |  |  |  |  |  |
|  | Dipteran larvae | 4 | 1.54 | >0.195 |  |  |  |  |  |  |
|  | Gastropoda  (*Melampus bidentatus*) | 4 | 1.21 | >0.305 |  |  |  |  |  |  |
|  | Hymenoptera | 4 | 0.30 | >0.741 |  |  |  |  |  |  |
|  | Isopoda | 4 | 1.09 | >0.344 |  |  |  |  |  |  |
|  | Nematoda | 4 | 1.13 | >0.330 |  |  |  |  |  |  |
|  | Pseudoscorpiones | 4 | 0.69 | >0.506 |  |  |  |  |  |  |
|  | "Others" | 4 | 1.07 | >0.349 |  |  |  |  |  |  |
