# Supplementary material for: Drivers of litter mass loss and faunal composition of detritus patches change over time
Source: Ecol Evol. 2021 Jun 23;11(14):9642–51. doi: 10.1002/ece3.7787 (PMC8293728; doi:10.1002/ece3.7787)
Supplement: Supplementary file 5 — Table S4 [file ECE3-11-9642-s006.docx]

Supplementary Table 4: ANOVA results for different time periods, explaining the dependency of different faunal taxa on the environmental parameters "habitat" and "litter" after 1 month (A), 6 months (B), and 12 months (C).

| **A: 1 month** | | SS | df | F | p |
| --- | --- | --- | --- | --- | --- |
| habitat | Acarina | 333.4 | 2 | 1.1 | 0.348 |
|  | Amphipoda  (*Orchestia gryllus*) | 0.4 | 2 | 1.1 | 0.350 |
|  | Arachnida | 1.1 | 2 | 2.7 | 0.076 |
|  | Chilopoda | 0.1 | 2 | 2.0 | 0.143 |
|  | Coleoptera | 4.1 | 2 | 1.7 | 0.186 |
|  | Collembola | 25.2 | 2 | 1.3 | 0.290 |
|  | Decapoda  (*Armases cinereum*) | 0.4 | 2 | 1.6 | 0.210 |
|  | Dipteran larvae | 94.8 | 2 | 2.6 | 0.085 |
|  | Gastropoda  (*Melampus bidentatus*) | 1981.6 | 2 | 19.2 | <0.001 |
|  | Hymenoptera | 0.3 | 2 | 1.0 | 0.356 |
|  | Isopoda | 1.4 | 2 | 2.5 | 0.088 |
|  | Nematoda | 0.1 | 2 | 2.0 | 0.143 |
|  | Pseudoscorpiones | 0.6 | 2 | 2.6 | 0.078 |
|  | "Others" | 116.0 | 2 | 3.0 | 0.054 |
| litter | Acarina | 6.1 | 1 | 0.1 | 0.843 |
|  | Amphipoda  (*Orchestia gryllus*) | 0.1 | 1 | 0.1 | 0.797 |
|  | Arachnida | 0.9 | 1 | 4.6 | 0.035 |
|  | Chilopoda | 0.0 | 1 | 0.0 | 1.000 |
|  | Coleoptera | 3.6 | 1 | 3.0 | 0.088 |
|  | Collembola | 1.7 | 1 | 0.2 | 0.683 |
|  | Decapoda  (*Armases cinereum*) | 0.1 | 1 | 0.4 | 0.529 |
|  | Dipteran larvae | 42.0 | 1 | 2.3 | 0.137 |
|  | Gastropoda  (*Melampus bidentatus*) | 24.5 | 1 | 0.5 | 0.493 |
|  | Hymenoptera | 0.2 | 1 | 1.4 | 0.242 |
|  | Isopoda | 0.0 | 1 | 0.0 | 1.000 |
|  | Nematoda | 0.0 | 1 | 0.0 | 1.000 |
|  | Pseudoscorpiones | 0.1 | 1 | 0.1 | 0.723 |
|  | "Others" | 37.6 | 1 | 1.9 | 0.165 |
| habitat X litter | Acarina | 526.3 | 2 | 1.7 | 0.192 |
|  | Amphipoda  (*Orchestia gryllus*) | 0.1 | 2 | 0.3 | 0.767 |
|  | Arachnida | 0.7 | 2 | 1.8 | 0.172 |
|  | Chilopoda | 0.0 | 2 | 0.0 | 1.000 |
|  | Coleoptera | 0.4 | 2 | 0.2 | 0.859 |
|  | Collembola | 9.5 | 2 | 0.5 | 0.622 |
|  | Decapoda  (*Armases cinereum*) | 0.1 | 2 | 0.4 | 0.672 |
|  | Dipteran larvae | 80.8 | 2 | 2.2 | 0.121 |
|  | Gastropoda  (*Melampus bidentatus*) | 91.6 | 2 | 0.9 | 0.416 |
|  | Hymenoptera | 0.4 | 2 | 1.4 | 0.254 |
|  | Isopoda | 0.1 | 2 | 0.2 | 0.857 |
|  | Nematoda | 0.0 | 2 | 0.0 | 1.000 |
|  | Pseudoscorpiones | 0.1 | 2 | 0.1 | 0.881 |
|  | "Others" | 42.9 | 2 | 1.1 | 0.331 |

| **B: 6 months** | | SS | df | F | p |
| --- | --- | --- | --- | --- | --- |
| habitat | Acarina | 1534.4 | 2 | 3.6 | 0.033 |
|  | Amphipoda  (*Orchestia gryllus*) | 0.9 | 2 | 3.0 | 0.056 |
|  | Arachnida | 18.5 | 2 | 1.9 | 0.151 |
|  | Chilopoda | 0.7 | 2 | 5.8 | 0.005 |
|  | Coleoptera | 1.2 | 2 | 3.3 | 0.042 |
|  | Collembola | 774.2 | 2 | 24.1 | <0.001 |
|  | Decapoda  (*Armases cinereum*) | 0.1 | 2 | 1.0 | 0.373 |
|  | Dipteran larvae | 753.7 | 2 | 3.2 | 0.047 |
|  | Gastropoda  (*Melampus bidentatus*) | 243.0 | 2 | 8.8 | <0.001 |
|  | Hymenoptera | 0.1 | 2 | 1.0 | 0.373 |
|  | Isopoda | 0.7 | 2 | 2.4 | 0.099 |
|  | Nematoda | 0.6 | 2 | 0.6 | 0.572 |
|  | Pseudoscorpiones | 0.4 | 2 | 2.1 | 0.126 |
|  | "Others" | 0.0 | 2 | 0.0 | 1.000 |
| litter | Acarina | 1027.6 | 1 | 4.8 | 0.032 |
|  | Amphipoda  (*Orchestia gryllus*) | 0.1 | 1 | 0.1 | 0.756 |
|  | Arachnida | 5.6 | 1 | 0.6 | 0.447 |
|  | Chilopoda | 0.1 | 1 | 0.2 | 0.630 |
|  | Coleoptera | 0.0 | 1 | 0.0 | 1.000 |
|  | Collembola | 62.3 | 1 | 3.9 | 0.053 |
|  | Decapoda  (*Armases cinereum*) | 0.1 | 1 | 1.0 | 0.321 |
|  | Dipteran larvae | 990.1 | 1 | 8.4 | 0.005 |
|  | Gastropoda  (*Melampus bidentatus*) | 17.0 | 1 | 1.2 | 0.271 |
|  | Hymenoptera | 0.1 | 1 | 1.0 | 0.321 |
|  | Isopoda | 0.1 | 1 | 0.9 | 0.357 |
|  | Nematoda | 0.1 | 1 | 0.1 | 0.744 |
|  | Pseudoscorpiones | 0.3 | 1 | 4.1 | 0.047 |
|  | "Others" | 0.5 | 1 | 6.6 | 0.012 |
| habitat X litter | Acarina | 388.2 | 2 | 0.9 | 0.407 |
|  | Amphipoda  (*Orchestia gryllus*) | 0.1 | 2 | 0.1 | 0.907 |
|  | Arachnida | 9.5 | 2 | 0.5 | 0.608 |
|  | Chilopoda | 0.1 | 2 | 0.2 | 0.792 |
|  | Coleoptera | 0.1 | 2 | 0.2 | 0.793 |
|  | Collembola | 64.2 | 2 | 2.0 | 0.144 |
|  | Decapoda  (*Armases cinereum*) | 0.1 | 2 | 1.0 | 0.373 |
|  | Dipteran larvae | 406.8 | 2 | 1.7 | 0.186 |
|  | Gastropoda  (*Melampus bidentatus*) | 21.0 | 2 | 0.8 | 0.471 |
|  | Hymenoptera | 0.1 | 2 | 1.0 | 0.373 |
|  | Isopoda | 0.2 | 2 | 0.9 | 0.427 |
|  | Nematoda | 1.1 | 2 | 1.2 | 0.322 |
|  | Pseudoscorpiones | 0.4 | 2 | 2.1 | 0.126 |
|  | "Others" | 0.0 | 2 | 0.0 | 1.000 |

| **C: 12 months** | | SS | df | F | p |
| --- | --- | --- | --- | --- | --- |
| habitat | Acarina | 16912.0 | 2 | 13.7 | <0.001 |
|  | Amphipoda  (*Orchestia gryllus*) | 0.1 | 2 | 1.0 | 0.373 |
|  | Arachnida | 21.8 | 2 | 20.0 | <0.001 |
|  | Chilopoda | 12.2 | 2 | 6.8 | 0.002 |
|  | Coleoptera | 3.1 | 2 | 1.7 | 0.196 |
|  | Collembola | 20694.8 | 2 | 27.8 | <0.001 |
|  | Decapoda  (*Armases cinereum*) | 0.1 | 2 | 1.0 | 0.373 |
|  | Dipteran larvae | 273.2 | 2 | 1.6 | 0.203 |
|  | Gastropoda  (*Melampus bidentatus*) | 138.2 | 2 | 7.1 | 0.002 |
|  | Hymenoptera | 5.9 | 2 | 4.9 | 0.010 |
|  | Isopoda | 55.4 | 2 | 2.4 | 0.096 |
|  | Nematoda | 3.4 | 2 | 1.8 | 0.165 |
|  | Pseudoscorpiones | 3.9 | 2 | 3.0 | 0.055 |
|  | "Others" | 1.0 | 2 | 1.3 | 0.284 |
| litter | Acarina | 882.0 | 1 | 1.4 | 0.236 |
|  | Amphipoda  (*Orchestia gryllus*) | 0.1 | 1 | 1.0 | 0.321 |
|  | Arachnida | 0.1 | 1 | 0.1 | 0.750 |
|  | Chilopoda | 1.7 | 1 | 1.9 | 0.177 |
|  | Coleoptera | 1.7 | 1 | 1.8 | 0.184 |
|  | Collembola | 88.9 | 1 | 0.2 | 0.627 |
|  | Decapoda  (*Armases cinereum*) | 0.1 | 1 | 1.0 | 0.321 |
|  | Dipteran larvae | 24.5 | 1 | 0.3 | 0.590 |
|  | Gastropoda  (*Melampus bidentatus*) | 1.1 | 1 | 0.1 | 0.734 |
|  | Hymenoptera | 0.1 | 1 | 0.1 | 0.761 |
|  | Isopoda | 16.0 | 1 | 1.4 | 0.240 |
|  | Nematoda | 0.7 | 1 | 0.8 | 0.390 |
|  | Pseudoscorpiones | 0.9 | 1 | 1.4 | 0.241 |
|  | "Others" | 0.1 | 1 | 0.3 | 0.573 |
| habitat X litter | Acarina | 114.3 | 2 | 0.1 | 0.912 |
|  | Amphipoda  (*Orchestia gryllus*) | 0.1 | 2 | 1.0 | 0.373 |
|  | Arachnida | 0.1 | 2 | 0.1 | 0.903 |
|  | Chilopoda | 3.4 | 2 | 1.9 | 0.164 |
|  | Coleoptera | 0.1 | 2 | 0.1 | 0.942 |
|  | Collembola | 1288.1 | 2 | 1.7 | 0.185 |
|  | Decapoda  (*Armases cinereum*) | 0.1 | 2 | 1.0 | 0.373 |
|  | Dipteran larvae | 40.1 | 2 | 0.2 | 0.787 |
|  | Gastropoda  (*Melampus bidentatus*) | 0.8 | 2 | 0.1 | 0.962 |
|  | Hymenoptera | 0.1 | 2 | 0.1 | 0.977 |
|  | Isopoda | 26.8 | 2 | 1.2 | 0.317 |
|  | Nematoda | 1.4 | 2 | 0.8 | 0.477 |
|  | Pseudoscorpiones | 1.9 | 2 | 1.5 | 0.239 |
|  | "Others" | 1.0 | 2 | 1.3 | 0.284 |
