# Supplementary material for: Drivers of litter mass loss and faunal composition of detritus patches change over time
Source: Ecol Evol. 2021 Jun 23;11(14):9642–51. doi: 10.1002/ece3.7787 (PMC8293728; doi:10.1002/ece3.7787)
Supplement: Supplementary file 6 — Table S5 [file ECE3-11-9642-s001.docx]

Supplementary Table 5: ANOVA results, explaining the effects of "habitat" (marsh, creek, woods), "litter" (oak, pine) and "fauna" presence of mesofauna versus mesofauna + macrofauna) on litter mass loss after 1 month (A), 6 months (B), 12 months (C), and 25 months (D).

| **A** | **SS** | **df** | **Mean Square** | **F** | **p** |
| --- | --- | --- | --- | --- | --- |
| Corrected Model | 75383.251 | 11 | 6853.023 | 3.431 | .001 |
| Intercept | 2318807.400 | 1 | 2318807.402 | 1160.931 | .000 |
| habitat | 9288.426 | 2 | 4644.213 | 2.325 | .107 |
| fauna | 74.740 | 1 | 74.740 | .037 | .847 |
| litter | 54845.639 | 1 | 54845.639 | 27.459 | .000 |
| habitat * fauna | 3897.468 | 2 | 1948.734 | .976 | .383 |
| habitat * litter | 1452.247 | 2 | 726.124 | .364 | .697 |
| fauna * litter | 3340.040 | 1 | 3340.040 | 1.672 | .201 |
| habitat * fauna * litter | 2975.194 | 2 | 1487.597 | .745 | .479 |
| Error | 115847.394 | 58 | 1997.369 |  |  |
| Total | 2510038.046 | 70 |  |  |  |
| Corrected Total | 191230.645 | 69 |  |  |  |
|  | | | | | |

| **B** | **SS** | **df** | **Mean Square** | **F** | **p** |
| --- | --- | --- | --- | --- | --- |
| Corrected Model | 87406.643 | 11 | 7946.058 | 2.392 | .017 |
| Intercept | 5890292.721 | 1 | 5890292.721 | 1773.379 | .000 |
| habitat | 11030.621 | 2 | 5515.311 | 1.660 | .199 |
| fauna | 931.430 | 1 | 931.430 | .280 | .599 |
| litter | 31579.932 | 1 | 31579.932 | 9.508 | .003 |
| habitat * fauna | 7911.238 | 2 | 3955.619 | 1.191 | .312 |
| habitat * litter | 10201.731 | 2 | 5100.866 | 1.536 | .224 |
| fauna * litter | 11454.252 | 1 | 11454.252 | 3.449 | .069 |
| habitat * fauna * litter | 15031.110 | 2 | 7515.555 | 2.263 | .114 |
| Error | 182682.933 | 55 | 3321.508 |  |  |
| Total | 6160382.297 | 67 |  |  |  |
| Corrected Total | 270089.576 | 66 |  |  |  |
|  | | | | | |

| **C** | **SS** | **df** | **Mean Square** | **F** | **p** |
| --- | --- | --- | --- | --- | --- |
| Corrected Model | 412191.462 | 11 | 37471.951 | 5.063 | .000 |
| Intercept | 14824008.433 | 1 | 14824008.433 | 2002.916 | .000 |
| habitat | 339157.454 | 2 | 169578.727 | 22.912 | .000 |
| fauna | 244.743 | 1 | 244.743 | .033 | .856 |
| litter | 1559.946 | 1 | 1559.946 | .211 | .648 |
| habitat * fauna | 5071.083 | 2 | 2535.542 | .343 | .711 |
| habitat * litter | 33722.728 | 2 | 16861.364 | 2.278 | .112 |
| fauna * litter | 20468.766 | 1 | 20468.766 | 2.766 | .102 |
| habitat * fauna * litter | 10798.726 | 2 | 5399.363 | .730 | .487 |
| Error | 429270.371 | 58 | 7401.213 |  |  |
| Total | 15665470.266 | 70 |  |  |  |
| Corrected Total | 841461.833 | 69 |  |  |  |
|  | | | | | |

| **D** | **SS** | **df** | **Mean Square** | **F** | **p** |
| --- | --- | --- | --- | --- | --- |
| Corrected Model | 1196649.000 | 11 | 108786.273 | 3.289 | .003 |
| Intercept | 18743176.606 | 1 | 18743176.606 | 566.650 | .000 |
| habitat | 695594.383 | 2 | 347797.191 | 10.515 | .000 |
| fauna | 2027.490 | 1 | 2027.490 | .061 | .806 |
| litter | 30937.201 | 1 | 30937.201 | .935 | .340 |
| habitat * fauna | 45625.502 | 2 | 22812.751 | .690 | .508 |
| habitat * litter | 369837.016 | 2 | 184918.508 | 5.591 | .007 |
| fauna * litter | 904.647 | 1 | 904.647 | .027 | .870 |
| habitat * fauna * litter | 16513.329 | 2 | 8256.665 | .250 | .780 |
| Error | 1256932.957 | 38 | 33077.183 |  |  |
| Total | 21196758.563 | 50 |  |  |  |
| Corrected Total | 2453581.957 | 49 |  |  |  |
|  | | | | | |
